# Longitudinal study highlights patterns of *Salmonella* serovar co-occurrence and exclusion in commercial poultry production

**DOI:** 10.3389/fmicb.2025.1570593

**Published:** 2025-07-16

**Authors:** Amy T. Siceloff, Doug Waltman, Christian E. Gunning, Sean P. Nolan, Pejman Rohani, Nikki W. Shariat

**Affiliations:** ^1^Department of Population Health, Poultry Diagnostic and Research Center, College of Veterinary Medicine, University of Georgia, Athens, GA, United States; ^2^Department of Microbiology, University of Georgia, Athens, GA, United States; ^3^Georgia Poultry Laboratory Network, Gainesville, GA, United States; ^4^Odum School of Ecology, University of Georgia, Athens, GA, United States; ^5^Nolan Integrated Pest Control and Management (NIPCAM) Group, Watkinsville, GA, United States; ^6^Center for the Ecology of Infectious Diseases, University of Georgia, Athens, GA, United States; ^7^Department of Infectious Diseases, University of Georgia, Athens, GA, United States; ^8^Department of Infectious Diseases, University of Georgia, Athens, GA, United States; ^9^Center for Food Safety, University of Georgia, Griffin, GA, United States

**Keywords:** *Salmonella*, multiserovar populations, poultry, CRISPR-SeroSeq, competitive exclusion

## Abstract

Recent advances in next-generation sequencing approaches have revealed that *Salmonella* often exists in multiserovar populations, with important implications for public health as time and resource constraints limit serovar characterization by colony-based isolation methods. It is important to characterize *Salmonella* population dynamics to then understand how the microbial ecology influences serovar evolution and thus, animal and human health outcomes. Chicken remains the leading source of foodborne *Salmonella* outbreaks in the U. S., despite reductions in contamination at the product level, underscoring the need for targeted control strategies. This study aimed to survey multiserovar *Salmonella* populations in broiler breeder flocks and monitor fluctuations throughout production. Deep serotyping was performed on environmental breeder samples collected over 2 years as part of a surveillance program. About 18% (104/568) of samples contained multiple serovars, with serovar Kentucky negatively associated with other serovars, often excluding them. Longitudinal sampling across two commercial complexes over 65 weeks included pullet and breeder farms. Environmental samples were collected via pre-moistened boot socks and rodent bait boxes, with on-farm rodents captured. *Salmonella* prevalence in pullet flocks was 17% (11/64), while 41% (135/330) of breeder samples were positive, peaking at 38 weeks of age. Rodents showed 35% (17/49) positivity in gastrointestinal samples and 9% (3/33) in bait station swabs, with six serovars identified, three of which were shared with flocks. Our cross-sectional and longitudinal *Salmonella* surveillance highlights the complexity of serovar interactions with further work required to elucidate the mechanisms of competitive exclusion.

## Introduction

*Salmonella* is a leading bacterial cause of foodborne illness in the United States, with an estimated 1.35 million infections, 26,500 hospitalizations, 420 deaths, and cost of illness of over $4 billion USD annually (United States Department of Agriculture - Economic Research Service, 2021; Centers for Disease Control and Prevention, 2024a,b). While *Salmonella* is ubiquitous in the environment, most *Salmonella* illnesses are foodborne, with more than 75% of outbreaks attributed to seven food categories (chicken, fruits, pork, seeded vegetables, other produce, beef, turkey) (Interagency Food Safety Analytics Collaboration, 2024). Importantly, chicken is considered the largest single food contributor, accounting for 19.7% of *Salmonella* outbreaks (Interagency Food Safety Analytics Collaboration, 2024). The use of post-harvest antimicrobial interventions in domestic broiler processing plants has supported a significant decrease in *Salmonella* incidence from 8.9% in 2016 to 6.5% in 2022, based on surveillance data collected in the contiguous states by the United States Department of Agriculture—Food Safety and Inspection Service (USDA—FSIS) (United States Department of Agriculture - Food Safety and Inspection Service, 2023); however, this has not been accompanied by a reduction in attribution of poultry in human salmonellosis cases (Centers for Disease Control and Prevention, 2024a,b; United States Department of Agriculture - Food Safety and Inspection Service, 2022). To maximize the success of post-harvest interventions, it is necessary to reduce the load of *Salmonella* entering the plant, which in turn requires increased pre-harvest control and surveillance (Bailey, 1993).

*Salmonella enterica* subsp. *enterica* is responsible for 99% of human salmonellosis, and it is comprised of over 1,500 different serovars, as identified by their lipopolysaccharide (O) and flagellar (H) antigens (Grimont and Weill, 2007; Lamas et al., 2018). In 2022, the five most commonly isolated serovars from human clinical cases in the United States were Enteritidis (2.7 cases per 100,000 population), Typhimurium (1.6), Newport (1.4), Javiana (0.9), and I 4,[5],12:i:- (0.6); these have also been the top five serovars annually since 2010 (Centers for Disease Control and Prevention, 2024a,b; Delahoy et al., 2022). Four of the five serovars are commonly isolated from food animal sources (poultry, beef, swine), while serovar Javiana is often attributed to fresh produce and thought to be associated with reptiles (Centers for Disease Control and Prevention, 2024a,b; Mukherjee et al., 2019; Rose et al., 2025). Different serovars pose different risks to public health based on their host restriction and adaptation (Uzzau et al., 2000), pathogenicity (Cheng et al., 2019), and propensity to carry antimicrobial resistance genes (Shah et al., 2016). Therefore, for meaningful food safety improvement, it is critical to identify which *Salmonella* serovars are present within a food product and to target mitigation against those that convey the greatest risk. For example, in poultry, serovars Kentucky and Enteritidis are commonly isolated; serovar Kentucky is not often responsible for human salmonellosis in the United States, while serovar Enteritidis is responsible for the largest number of cases each year (Centers for Disease Control and Prevention, 2024a,b).

Poultry production begins with pullet flocks, consisting of sexually immature chickens that are raised in single sex houses until ~21 weeks of age. At this point, pullet flocks are divided and transferred to breeder farms, where fertilized eggs will then become broiler chickens that are grown for five to 9 weeks before slaughter. Breeder flocks remain in production until ~65 weeks, the average breeder hen will lay around 180 eggs with peak production between 28 and 32 weeks of age (McDaniel, 2021). In the United States, commercial chicken production is vertically integrated, with each stage of production maintained within a single complex that belongs to a single company (integrator). Poultry disease management (e.g., vaccination) is usually performed at the complex level; this also extends to *Salmonella* controls (e.g., vaccination, water acidification, or use of litter amendments or pre- and probiotics). For a single integrator, management strategies differ from complex-to-complex, depending on the *Salmonella* risks and serovars detected at processing. Each complex encompasses both live production (breeder flocks, hatchery, broiler flocks, and feed mill) and processing (slaughter and distribution) stages. This allows for greater control and coordination across the entire supply chain, leading to more efficient production and distribution, and improved food safety and quality control. Vertical integration also supports greater biosecurity control as integrators can limit pathogen introduction to flocks, but subsequently provides the opportunity for vertical transmission of existing pathogens from parent to progeny.

To add further complexity to poultry production, multiple *Salmonella* serovars can exist within a population (Thompson et al., 2018; Rasamsetti et al., 2022; Siceloff et al., 2022; Obe et al., 2023; Rasamsetti and Shariat, 2023; Richards et al., 2024). However, the conventional methods of *Salmonella* culturing typically only identify the most abundant serovar within a population or the serovar that can best outcompete others under certain enrichment conditions (Gorski et al., 2024). For many laboratories, time and resource constraints often necessitate selecting only one colony from an indicator agar plate. For a 95% probability of identifying two serovars from a sample, six colonies must be isolated and the two serovars must exist in equal proportions (Cason et al., 2011). This limitation can be mitigated in part where careful attention is spent to select a small number of colonies that have different colony morphologies. Alternatively, molecular-based deep serotyping, such as CRISPR-SeroSeq, can provide greater resolution of *Salmonella* populations by identifying multiple serovars that co-occur within a sample. Previous studies on *Salmonella* complexity in poultry have demonstrated that 32% of *Salmonella-*positive samples from breeders and 57% of *Salmonella*-positive broiler houses contain more than one serovar (Siceloff et al., 2022; Obe et al., 2023). At processing, 48 and 7.9% of *Salmonella-*positive carcasses at hot rehang and post-chill, respectively, have multiserovar populations (Richards et al., 2024). Our previous study sought to compare the serovars isolated from live production and processing operations to better understand *Salmonella* transmission dynamics in the poultry industry, but the discrepancies between serovars identified at both stages further highlighted the need for high-resolution surveillance to elucidate transmission patterns (Siceloff et al., 2022).

Previous work has demonstrated that both vertical and horizontal transmission of *Salmonella* occurs within a poultry complex, as matching subtypes were isolated from breeder farms and their subsequent broilers both on farm and at processing (Byrd et al., 1998; Liljebjelke et al., 2005; Hannah et al., 2011; Gast et al., 2014; Crabb et al., 2018; Lei et al., 2020). Some serovars, such as Enteritidis, can enter the fertilized egg, which then leads to colonization of the chicks and spread among flocks as the birds share a common environment for several weeks of production (Gast and Beard, 1990; Humphrey et al., 1991; Keller et al., 1995; Miyamoto et al., 1997; Guard-Petter, 2001). Additionally, *Salmonella* may be present on the exterior of the eggshell through fecal contamination (Gantois et al., 2009). Because breeder flocks colonized with *Salmonella* can be a source of downstream *Salmonella* in broiler flocks, integrators have focused on *Salmonella* monitoring and control in their breeder flocks, with elective testing in pullet and breeder flocks around 16 and 42 weeks, respectively.

Effective *Salmonella* controls in breeders include vaccination and increased biosecurity. There are three types of *Salmonella* vaccinations used in broiler production the United States: (1) commercial live attenuated vaccines against serovar Typhimurium; (2) a commercial killed vaccine against serovar Enteritidis; and (3) autogenous (killed) vaccines that are generated against specific serovars and are generally limited for use within a single complex. These three types of vaccine are most frequently used in combination. A recent survey of 23 complexes in Georgia showed that all complexes were vaccinating their breeders against *Salmonella*, with 92% using a live-attenuated Typhimurium vaccine (Georgia Poultry Laboratory Network, 2025). Because delivery of killed vaccines necessitates individual bird handling and the multiplication of broilers is so large, use of these vaccines is typically restricted to breeders. It has been observed that live attenuated vaccines can provide cross-protection to animal hosts against additional serovars other than the original vaccine strain, though the efficacy varies across isogenic groups and serogroups (Tennant et al., 2015; Hofacre et al., 2021; Bearson et al., 2024).

Increased on-farm biosecurity can also help prevent *Salmonella* transmission. Best biosecurity practices include not sharing equipment between farms or cleaning equipment before use, disinfecting vehicles before entering the property, use of disposable boot covers and sanitizing footbaths prior to entering a house, controlling rodent and insect populations, maintaining dry litter, and ensuring that the houses are structurally intact to prevent any interactions with wildlife (United States Department of Agriculture - Animal and Plant Health Inspection Service, 2024a,b). In addition to human activity, rodents and insects may serve as disease carriers and introduce pathogens, such as *Salmonella*, to poultry flocks (Henzler and Opitz, 1992; Davies and Wray, 1995; Goodwin and Waltman, 1996; Garber et al., 2003; Meerburg and Kijlstra, 2007; Lapuz et al., 2012; Trampel et al., 2014; Dale et al., 2015; Raufu et al., 2019; Smith et al., 2022).

The overall goal of this study was to measure the changes in *Salmonella* prevalence and serovar population dynamics during broiler breeder production and determine the incidence of multiserovar populations in breeder flocks. Additionally, we sought to assess if *Salmonella* transmission was occurring between breeder flocks and rodent populations. The study was accomplished in two parts. First, to investigate broad *Salmonella* patterns in breeder flocks, we performed deep serotyping on 568 blinded samples collected from breeder flocks over a two-year period. Second, to more finely assess *Salmonella* prevalence and serovar dynamics in breeders, we collected monthly samples from eight breeder flocks (13 different houses) and their source pullet flocks as well as rodents from the corresponding farms over one full production cycle (65 weeks) and used deep serotyping to assess *Salmonella* populations. Our findings highlight the importance of on-farm biosecurity and reveal, for the first time, patterns of serovar co-occurrence and exclusion.

## Materials and methods

### Longitudinal breeder flock sample collection and *Salmonella* culturing

Across two commercial complexes (Complexes 1 and 2), 15 pullet (five farms) and 13 breeder houses (seven farms) were sampled over a 65-week production period. Pullets were sampled at weeks 14 and 21, then breeders sampled monthly, apart from weekly sampling during peak production (29–31 weeks). Prior to flock placement, the empty, cleaned (slats scraped and cleaned, water and feed receptacles cleaned, and fresh shavings placed) breeder houses were sampled to determine if there was any residual *Salmonella* contamination from the previous flock. Complex 2 Farm 1 (2–1) was not sampled prior to placement as the birds had been moved in early (sampling occurred within a few hours of placement) but it had been cleaned out prior. Two pre-moistened boot sock pairs (Romer Labs, Newark, DE) were collected from each house, walking between the feed and water lines on both sides of the scratch (pullets) or on the slats (breeders), and cultured for *Salmonella* (n = 394). Rodents (mice (*Mus musculus*) plus roof (*Rattus rattus*) and Norway (*Rattus norvegicus*) rats; n = 355 carcasses across 49 composite samples) were captured from breeder farms by an integrated pest management company and tested for *Salmonella*, along with bait station swabs (*n* = 33).

All samples were stored on ice during transportation back to the laboratory (between 1 and 4 h). 200 mL of buffered peptone water (BPW; Neogen, Lansing, MI) was added to each boot sock and homogenized with a Seward stomacher (Stomacher^®^ 400 Circulator Lab Blender, Bohemia, NY) for two minutes at 230 rpm. Following Hygiena’s protocol for *Salmonella* enrichment and quantification (data not shown), 60 mL of BPW was transferred to 60 mL of pre-warmed MP media (Hygiena, Camarillo, CA) with novobiocin (40 mg/L; Thermo Scientific Chemicals, Waltham, MA) and incubated shaking at 42°C for 10 h. Subsequently, 1 mL of culture was inoculated into 10 mL of tetrathionate broth (TT; Hardy Diagnostics, Santa Maria, CA), then incubated at 37°C for 20–24 h. For the rodent samples, 200 mL BPW with novobiocin (40 mg/L) was added to the removed gastrointestinal (GI) tract, homogenized, and incubated at 42°C for 20–24 h, then 1 mL and 0.1 mL of culture were added to 10 mL of TT and Rappaport-Vassiliadis (RV; Hardy Diagnostics, Santa Maria, CA) broth, respectively, and incubated at 37°C for 20–24 h. Following selective enrichment, all cultures were streaked onto xylose lysine tergitol-4 (XLT-4; Hardy Diagnostics, Santa Maria, CA) plates, then incubated at 37°C for 24–48 h. Any presumptive *Salmonella* colonies were restreaked onto Luria-Bertani (LB; Hardy Diagnostics, Santa Maria, CA) agar, then confirmed with serum agglutination (BD Difco, Franklin Lakes, NJ). All enrichments were pelleted via centrifugation at 14,000 rpm for 3 min, then stored at −20°C.

### Georgia poultry laboratory network sample collection

Several commercial poultry integrators participate in a routine *Salmonella* surveillance program through the Georgia Poultry Laboratory Network (GPLN), where samples are collected from breeder flocks at approximately 16 weeks (pullets; pre-egg production) and 40 weeks (post-peak egg production). In addition to integrators with conventional broiler breeder flocks, hatching egg companies maintain breeder flocks and these must be tested for *Salmonella* every 30 days in accordance with the National Poultry Improvement Plan (NPIP). The data in this study includes five different hatching egg companies, 11 different integrators, and six breeding companies. For each breeder flock, up to six samples are submitted in a single accession, typically with two boot socks in Whirl-Pak bags collected from the slats on each side of the house (left, right), two boot socks through the middle scratch area, and two miscellaneous environment samples (e.g., egg belt or ventilator fan swabs); only the four standardized samples were considered for this study. The metadata affiliated with each sample includes age of flock (if available), coded company name (to maintain blinded study), sample type, and date submitted. As part of a previous study, a subset of these samples (*n* = 134) was analyzed in comparison to processing plant samples and the results were published (Siceloff et al., 2022).

Between 125 and 150 mL of tetrathionate (TT) enrichment broth was added to each sample, followed by incubation at 37°C for 20 to 24 h. The bags were gently mixed, and 100 μL of enrichment was inoculated into a modified semisolid Rappaport-Vassiliadis (MSRV: Oxoid, ThermoFisher, Waltham, MA) agar plate then incubated at 42°C. The plates were checked at 24 and 48 h and presumptive *Salmonella* growth was transferred onto two types of agar: brilliant green (BG) agar containing novobiocin and xylose lysine tergitol-4 (XLT-4). These were incubated at 37°C for 20 to 24 h, and four presumptive *Salmonella* colonies were selected for further characterization. For each colony, *Salmonella* was confirmed by biochemical identification using the Vitek system (BioMerieux) and serogrouped by conventional serum agglutination (BD Difco, Fisher Scientific, Atlanta, GA; Remel, Lenexa, KS; and SSI Diagnostics, Cedarlane, Burlington, NC). Isolates from boot sock samples with unique serogroups per accession (flock) were then serotyped at GPLN using the Luminex xMap molecular assay (Luminex, Austin, TX).

For the days that we collected samples, we selected one *Salmonella*-positive boot sock sample from each breeder flock submitted to GPLN from July 2020 to June 2022 to complete CRISPR-SeroSeq. The samples for the study were collected on 1 day per week, shifting by 1 day each week to avoid bias of oversampling companies who may regularly submit on the same day weekly. The number of sample collection days differs per month, and the sample number is variable (higher sample numbers later in the week than earlier in the week), so our data set is not uniform across the months but contains at least one sampling day per month over 24 months. From the main sample set, a subset of samples submitted on the same day from breeder flocks on the same farm were chosen to complete a paired house study to measure the rate of on-farm *Salmonella* transmission. For each *Salmonella*-positive sample, the overnight TT enrichment cultures were briefly vortexed, and 1 mL of each was transferred into microcentrifuge tubes, centrifuged at 2,000 × g for 10 min, and stored at −20°C until later use.

### CRISPR-SeroSeq

Total genomic DNA was isolated from the *Salmonella*-positive culture pellets using the Genome Wizard kit (Promega, Madison, WI, United States), according to the manufacturer’s instructions, then DNA was resuspended in 200 μL of molecular grade water and stored at −20°C. For the 134 samples that were previously analyzed, and for the 394 samples from the longitudinal study, the CRISPR-SeroSeq libraries were generated using a 2-step PCR process, with the first PCR targeting the conserved CRISPR direct repeat sequences and the second PCR adding Illumina adaptors and index sequences as described (Thompson et al., 2018). For the remaining 434 samples from GPLN, a 1-step PCR was used with the Illumina adaptors and index sequences incorporated into the same primers as the target sequence, as described (Richards et al., 2024). The extracted DNA for each *Salmonella*-positive enrichment was used as template for the reaction, and the PCR products were visualized on a 2% agarose gel to confirm amplification. Following purification with AMPure XP beads (Beckman-Coulter, Indianapolis, IN, United States), the samples were pooled at approximate equimolar ratios and the resulting library was sequenced (150 cycles, single read, Illumina, San Diego, CA, United States). The sequence data has been uploaded to NCBI SRA as part of BioProject PRJNA1204137.

Sequences were analyzed using the CRISPR-SeroSeq pipeline by means of an R script (version 4.04) that utilizes a local alignment search tool (Altschul et al., 1990) to match experimental reads to a curated database containing the complete CRISPR profiles for over 150 serovars. BLAST matches with 100% coverage and identity are recorded on an Excel sheet and the relative frequency was calculated with unique spacer reads corresponding to each serovar (Siceloff et al., 2022). Serovars with a relative frequency greater than 0.5% were included in the analysis for all individual samples. Where a spacer was shared between two serovars present in a sample, the unique spacer read counts for each serovar were used to proportionally allocate the reads of the shared spacer to the two serovars. The CRISPR sequences are insufficient to distinguish between serovars Durban, Kokomele, Panama, Pomona, and Reading II, and in this instance, all five serovars are listed. Many *Salmonella* serovars are polyphyletic (Worley et al., 2018; Cherchame et al., 2022), and these evolutionary patterns are reflected in the CRISPR sequences (Shariat et al., 2013; Nguyen et al., 2018; Vosik et al., 2018). Where this occurs (e.g., Montevideo I and II), we have attributed a I, II, or III to indicate different lineages of a single serovar. For the longitudinal study, serovar populations were normalized across both boot socks pairs collected from one house on a single sampling visit.

### Statistical analysis

All statistical analyses were conducted with R (version 4.2.3). While the longitudinal dataset includes repeated measures from the same flocks over time, analyses were conducted under the assumption of independence between observations. The paired house subset from the GLPN dataset was normalized using the DESeq2 package^1^ to adjust the read counts per sample based on the size factors present.

## Results

**Figure 1:**
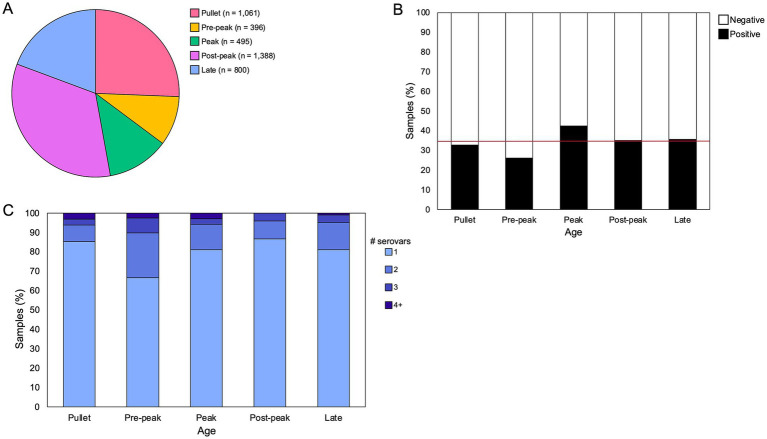
*Salmonella* prevalence and presence of multiserovar populations by age. **(A)** Proportion of submitted breeder samples per age class from July 2020 to June 2022 on predetermined sample collection days (*n* = 4,140). Categories are defined as – pullet: 0–21 weeks, pre-peak: 21–28 weeks, peak: 28–35 weeks, post-peak: 35–50 weeks, late: after 50 weeks. **(B)** Frequency of submitted samples that were positive or negative for *Salmonella* in each age class, with an observed relationship between age and prevalence (*p* < 0.00005, Chi-squared Test). The red horizontal line denotes the average prevalence across the sample set (34.7%). **(C)** Distribution of serovars/sample identified through deep serotyping of 568 *Salmonella*-positive samples. Counts of multiserovar populations across age classes are varied but not statistically significant (*p* = 0.06, Fisher’s Exact Test).

From July 2020 to June 2022, a total of 4,485 samples from 1,421 breeder flocks were submitted to the Georgia Poultry Laboratory Network (GPLN) on our sample collection days, and 35% (1,581/4485) of these were *Salmonella*-positive. Flock age information was provided for 92% (4,140/4485) of the samples (Figure 1A). One-quarter of the submitted samples were from pullet flocks under 21 weeks of age. Within breeder flocks, most submitted samples were after peak production, between 35 and 50 weeks (34%; 1388/4485). The high proportions at these two time ranges corresponds to the participation of many companies in screening their pullet and breeder flocks around 16 and 42 weeks, respectively. *Salmonella* prevalence was highest in flocks aged 28–35 weeks (42%; 210/495), the time frame that corresponds to breeder peak production. Prevalence was lowest in flocks aged 21–28 weeks (26%; 104/396), and there was an observed relationship between age and prevalence such that the prevalence within each age class was not due to random chance (Figure 1B; *p* < 0.00005, Chi-squared Test).

To assess the overall serovar diversity throughout the GPLN sample set, one *Salmonella*-positive sample was selected from each flock on each collection day to complete deep serotyping using CRISPR-SeroSeq, for a total of 568 samples analyzed with 22 companies represented (Fig. S1). A total of 38 serovars were identified, including 16 serovars and one untypeable serovar that were each found in at least five different samples (Table 1; Supplementary Table S1). There was an average of 1.3 serovars per sample, with a maximum of nine serovars identified from one boot sock. About one-fifth (18%; 104/568) of samples contained more than one serovar (Figure 1C). The measured frequency of multiserovar populations was the greatest in flocks aged 21–28 weeks (pre-peak production; 33%; 13/39), and lowest in pullet flocks (15%; 19/130) and flocks aged 35–50 weeks (post-peak production; 13%; 26/196). However, there was not a significant difference in the multiserovar populations recorded across the age classes (*p* = 0.06, Fisher’s Exact Test).

**Table 1:** CRISPR-SeroSeq summary results of Salmonella-positive samples from GPLN (*n* = 568).

Serovar^a^	Frequency^b^	Present (%)^c^	Alone (%)^d^	Major (%)^e^	Average relative frequency (%)^f^	Months^g^	Companies^h^
Kentucky I	462	81.3	86	65	94	23	20
Cerro	43	7.6	30	57	65	10	5
Mbandaka	34	6	12	17	30	15	7
**Typhimurium**	25	4.4	32	41	62	14	11
Liverpool	21	3.7	29	53	58	11	6
**Infantis**	19	3.3	16	12	38	11	6
Alachua	17	3	47	11	61	6	2
Senftenberg II	12	2.1	8	18	36	8	6
Tennessee	9	1.6	11	25	36	5	5
**Enteritidis**	8	1.4	62	0	72	5	7
Uganda	8	1.4	12	57	49	5	1
**Montevideo I**	6	1.1	0	17	18	4	3
**Montevideo II**	6	1.1	0	0	4	3	4
Agona	5	0.9	40	0	45	3	3
Altona	5	0.9	20	25	39	5	2
Anatum	5	0.9	20	25	42	2	3
Untypeable	5	0.9	20	0	32	3	2

a Only serovars present in five or more samples were included (n = 17), including five serovars of clinical importance (bolded), that are most often causing outbreaks (Center for Disease Control and Prevention BEAM Dashboard). The suffixes (-I, -II, -III) for some serovars refer to polyphyletic lineages.

b Indicates the total number of samples each serovar was found in.

c Indicates the total percentage of samples each serovar was found in.

d Indicates how often a serovar was the single serovar in a sample.

e Indicates the frequency in which the serovar was present at a higher relative frequency in a mixed population of multiple serovars.

f This was calculated across all “present” samples.

g Indicates how many months (*n* = 24) each serovar was identified in.

h Indicates how many companies (*n* = 22) each serovar was identified from.

**Figure 2:**
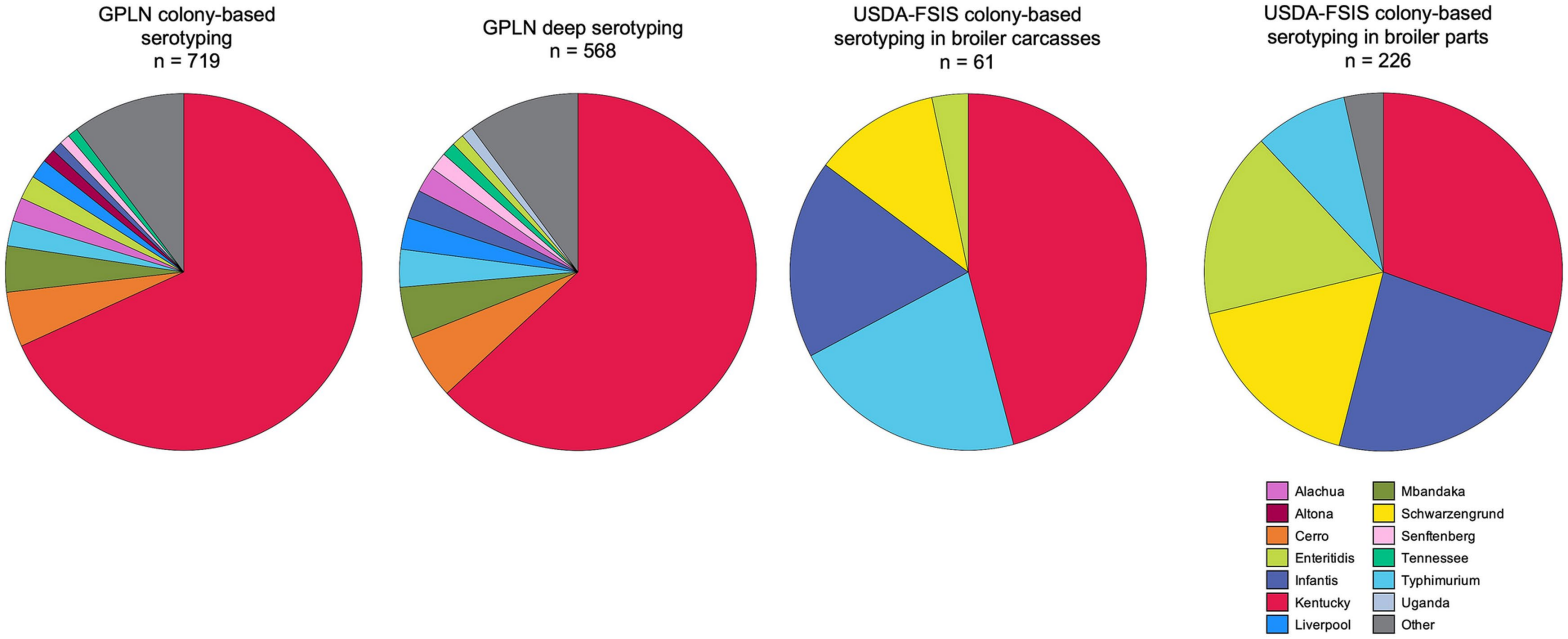
*Salmonella* serovars in breeder flocks and broiler carcasses and parts at processing collected in Georgia from August 2020 to June 2022. The two pie charts on the left represent serotyping results from environmental samples sent to GPLN; only the 10 most frequently identified serovars are reported. The leftmost pie chart contains the CRISPR-SeroSeq results for the GPLN subset analyzed in this study, while the second pie chart includes all samples traditionally serotyped (i.e., colony-based) during the study dates. The two pie charts on the right indicate traditional serotyping results from samples routinely collected at domestic processing establishments by USDA-FSIS, separated by sample type of parts (i.e., legs, breasts, wings) and whole carcasses; only the 5 most frequently identified serovars are reported (Raw Poultry Sampling, https://www.fsis.usda.gov/news-events/publications/raw-poultry-sampling, accessed 5 April 2023).

To compare the most common serovar identities between pre- and post-harvest, we downloaded the *Salmonella* regulatory sampling results from domestic poultry processing establishments in Georgia as collected by USDA – FSIS for the same period as the study (2020–2022) (Figure 2), and expanded the GPLN dataset to include all boot sock samples with conventional serotyping information (*n* = 719). Serovar Kentucky was the most abundant serovar across both GPLN and FSIS datasets, with a marked decrease of the second most abundant serovar in the GPLN dataset (serovar Cerro; 5.1% by colony serotyping) but not FSIS (serovars Infantis (24% in parts) and Typhimurium (21% in carcasses)). Alternatively, serovar Infantis was found 3.3% (19/568) and 0.97% (7/719) of breeder samples through deep serotyping and conventional serotyping, respectively. Similarly, serovar Typhimurium was identified in 4.4% (25/568) of samples with deep serotyping and 2.4% (17/719) of conventionally serotyped breeder samples. Notably, serovar Schwarzengrund was not present in the top 10 serovars isolated from breeder flocks while it was often found at processing. Overall serovar diversity was greater in the pre-harvest samples, with similar profiles observed from both deep serotyping and conventional serotyping due to the selection and typing of multiple colonies for isolation at GPLN, according to the National Poultry Improvement Plan (NPIP) *Salmonella* isolation protocols (United States Department of Agriculture - Animal and Plant Health Inspection Service, 2024a,b).

**Figure 3:**
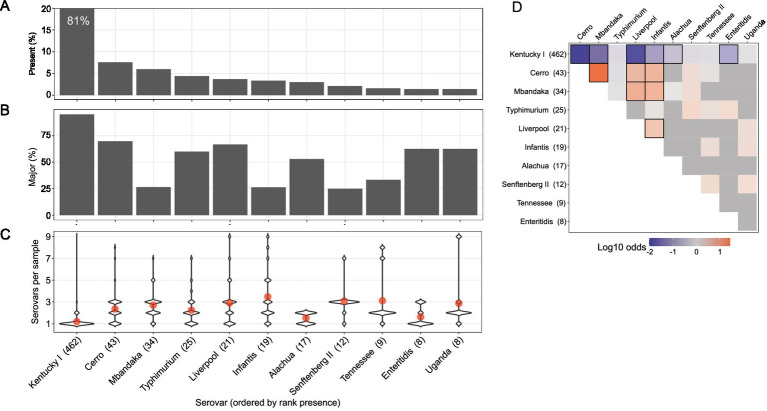
Multiserovar population dynamics vary by serovar. Only the top 10 most present serovars from the dataset are shown, with serovars Enteritidis and Uganda tied in 10th place. **(A)** Top bar graph indicates the total sample percentage that each serovar was identified in, with only serovars present in 5 or more samples being displayed. **(B)** Percent of samples in which each serovar was present and considered major, or most abundant. **(C)** Violin plot shows number of serovars per sample (Y) when a specific serovar (X, ordered by rank presence) is present. Red dots denote mean number of serovars per sample (among samples containing each indicated serovar). **(D)** Log_10_ odds ratio of serovar co-occurrence. Row labels show number of samples where serovar was present. Red shading shows positive association while blue shading shows negative association. Black outlines indicate cells with FDR < 0.05.

From the deep serotyping results of the GPLN dataset, serovar Kentucky was most often identified as the major serovar within a sample (as defined by the relative frequency): in 86% (396/462) of samples where it was detected, it was the sole serovar and in samples where it co-occurred with another serovar (n = 66), it was the major serovar in 65% (43/66) of these (Figure 3A). The average relative frequency of serovar Kentucky when it was present was 94%, as determined by calculating the mean of relative frequencies in each corresponding sample with deep serotyping results (Table 1). Although at a significantly lower incidence, serovar Cerro was the second most prevalent serovar detected (n = 43; 7.6%) followed by serovar Mbandaka (n = 34; 6.0%). When comparing the presence versus majority of the top ten serovars in our dataset, some serovars displayed a higher overall frequency across the samples but lower relative frequency within samples (Figures 3A,B). For example, serovar Mbandaka was present in 6.0% of samples (34/568) but was major or alone in 27% (9/34) of these and at an average relative frequency of 30% (Figures 3A,B and Table 1).

To observe any relationships between serovar identity and overall serovar complexity within a sample, we compared the distribution of serovars per sample for the ten most abundant serovars in the dataset (Figure 3C). Serovar Kentucky was most often found as the only serovar within a sample (red dot in Figure 3C; mean = 1.2 serovars per sample when serovar Kentucky is present). Alternatively, serovars Cerro and Mbandaka were often found within samples containing multiple serovars (mean number of serovars per sample of 2.3 and 2.7, respectively). This trend was observed with five of the other top ten serovars as well, with the exception of serovars Alachua and Enteritidis which were most often detected in samples with low serovar complexity. Of the top 10 most frequently detected serovars, serovar Infantis was often detected as a member of complex multiserovar samples (mean number of serovars per sample = 3.5) and was infrequently found alone (16%; 3/19). To determine if there was a pattern of serovar co-occurrence, we calculated the pairwise odds ratio of co-occurrence for the top ten serovars (Figure 3D). To account for multiple comparisons, we controlled the false discovery rate (FDR) at *α* = 0.05 (Benjamini and Yekutieli, 2001). In concordance with previous observations, serovar Kentucky has a significantly negative odds ratio of being identified with other serovars (FDR < 0.05). Serovars Cerro, Mbandaka, Liverpool, and Infantis all had significantly positive odds ratios, indicating that they are more likely to co-occur with each other. This is consistent with the frequent finding of these four serovars in multiserovar populations.

**Figure 4:**
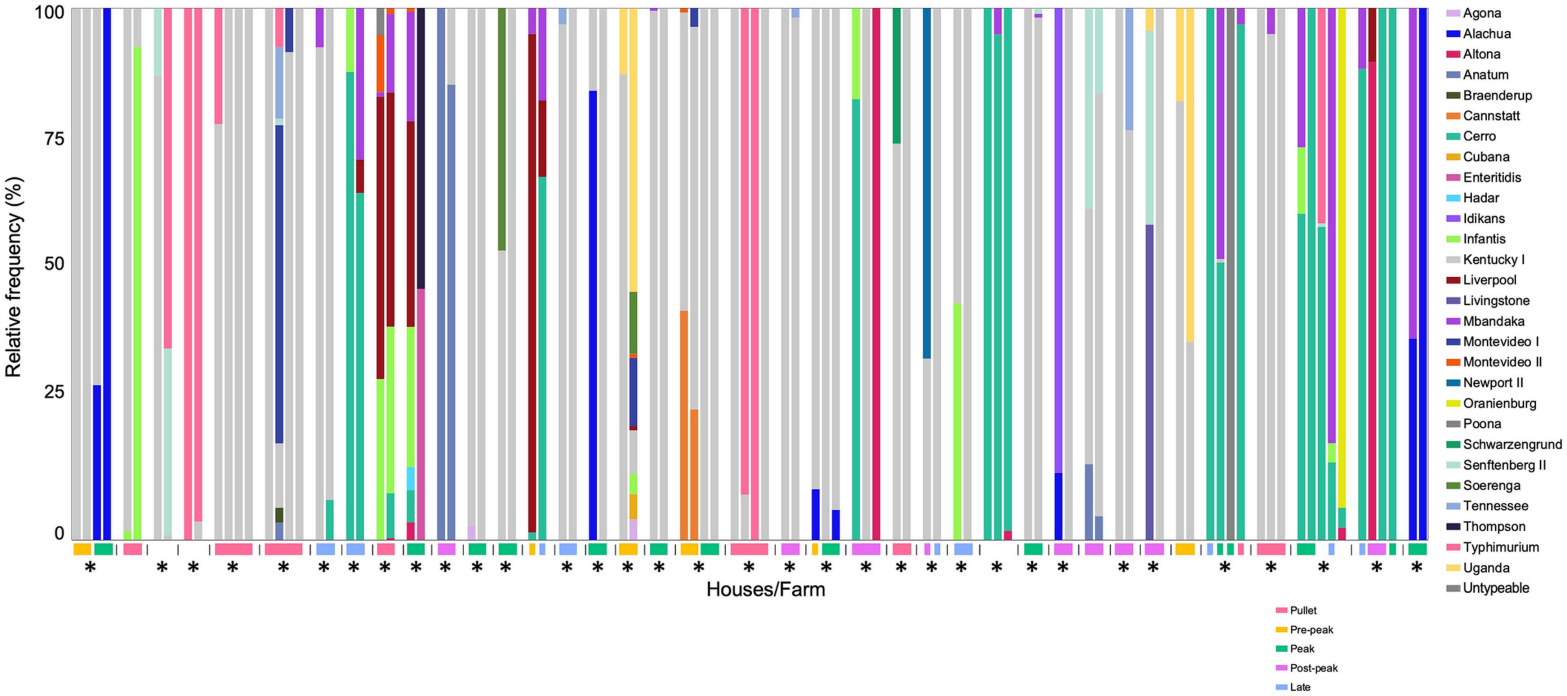
Multiserovar populations observed among multiple farms with several pullet/breeder houses. Deep serotyping results for farms containing multiserovar populations (38/129) are shown, with samples from the same farm indicated by the vertical lines, age of flock represented by the colored rectangle, and farms with at least one serovar not shared among all the houses are denoted with an asterisk. Flocks without provided age information are shown without a corresponding-colored rectangle. Pullet: 0–21 weeks, pre-peak: 21–28 weeks, peak: 28–35 weeks, post-peak: 35–50 weeks, late: after 50 weeks.

To effectively control *Salmonella*, it is necessary to not only identify all serovars present but also to recognize the sources and transmission patterns of *Salmonella*. Thus, to determine the level of on-farm *Salmonella* transmission, we chose a subset of samples submitted to GPLN representing multiple breeder houses on the same farm that were collected on the same day; this subset is not mutually exclusive from the main GPLN dataset due to the instances where a paired house sample was also the representative sample for the flock accession. In total, there were 322 boot sock samples, each representing a single breeder house across 129 farms. The number of houses on each farm ranged from two to eight houses. The majority of these samples (82%; 265/322) contained only a single serovar., which, as expected, was predominantly serovar Kentucky. There was an average of 1.3 serovars per sample, with a total of 38 farms that contained at least one multiserovar population (i.e., on a single boot sock) (Figure 4). In 34 of these 38 farms, there was at least one serovar present that was absent in another house on the same farm. Additionally, there were 10 farms comprised of single serovar populations where at least one house contained a separate serovar from the rest (Supplementary Figure S2). From the entire paired house dataset (129 farms, 322 houses), the Bray–Curtis dissimilarity was calculated pairwise for all houses on a farm and then averaged to determine the similarity of on-farm populations; 70% (90/129) of farms contained similar populations (Bray-Curtis: 0–0.3), with the remaining farms consisting of moderate similarity (23/129; Bray-Curtis: 0.3–0.7) or dissimilar populations (16/129; Bray-Curtis: 0.7–1). Additionally, an ANOVA model indicated the Shannon diversity index based on present serovars varied with age class (*p* < 0.005). Collectively, these results demonstrate that serovar complexity may be influenced by the presence of multiple houses on one farm but also depends on the age of the flock.

**Figure 5:**
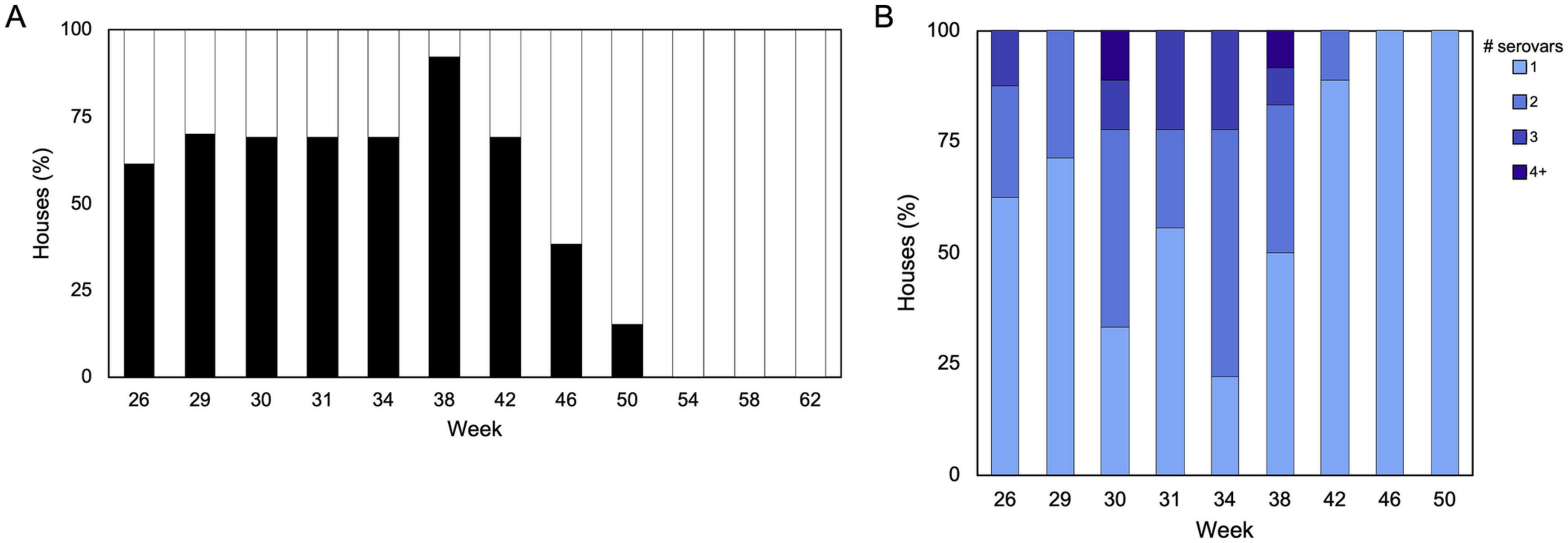
*Salmonella* prevalence and serovar complexity throughout one breeder production cycle. **(A)** Proportion of breeder houses (*n* = 13) that were positive or negative for *Salmonella* during each sampling week (*n* = 12). One farm was inaccessible during week 29, so only 10 houses are represented at that timepoint. **(B)** Distribution of single and multiserovar populations identified in each house. Spearman’s rank correlation suggests that there is a stronger association between prevalence and complexity [r_s_ = 0.79, *p* = 0.01 (Spearman’s rank correlation rho)].

The high-resolution viewpoint of *Salmonella* populations in breeder flocks provided above is useful to identify broad patterns but we next sought to more closely investigate whether *Salmonella* incidence and serovar population dynamics change through the lifetime of individual flocks. For this longitudinal study, 394 boot sock samples were collected from 15 pullet houses (P1-P15, across five flocks) and 13 breeder houses (B1-B13, across eight flocks; sourced from the 15 pullet houses) across two commercial broiler breeder complexes (1 and 2) over a 65-week production period (Supplementary Figure S3). Importantly, Complex 1 employed an integrated pest control service to control rodent and insect populations, while Complex 2 relied on farm staff. Sampling was increased during peak production (i.e., when the hens are laying the most eggs; weeks 29–31 in this study) because we hypothesized that the birds would be shedding more *Salmonella* during this time due to stress; however, we found that *Salmonella* prevalence peaked at 38 weeks (Figure 5A). Overall, 37% (146/394) of samples were *Salmonella*-positive, with a prevalence of 17% (11/64) and 41% (135/330) from pullet and breeder samples, respectively. Only the pullet houses in Complex 2 were positive for *Salmonella* (6/7 houses), while 92% (12/13) of breeder houses across both complexes were *Salmonella*-positive at least one sampling point (Supplementary Figure S3). Importantly, only two breeder flocks were positive at week 50 (flocks B1 and B2, which were on the same farm), and no flocks were positive after this time. We observed a parabolic curve of the prevalence over the duration of the study, such that the prevalence increased until week 38, and this was accompanied by a corresponding increase in multiserovar populations (Figure 5B; r_s_ = 0.79; *p* = 0.01, Spearman’s rank correlation r).

**Figure 6:**
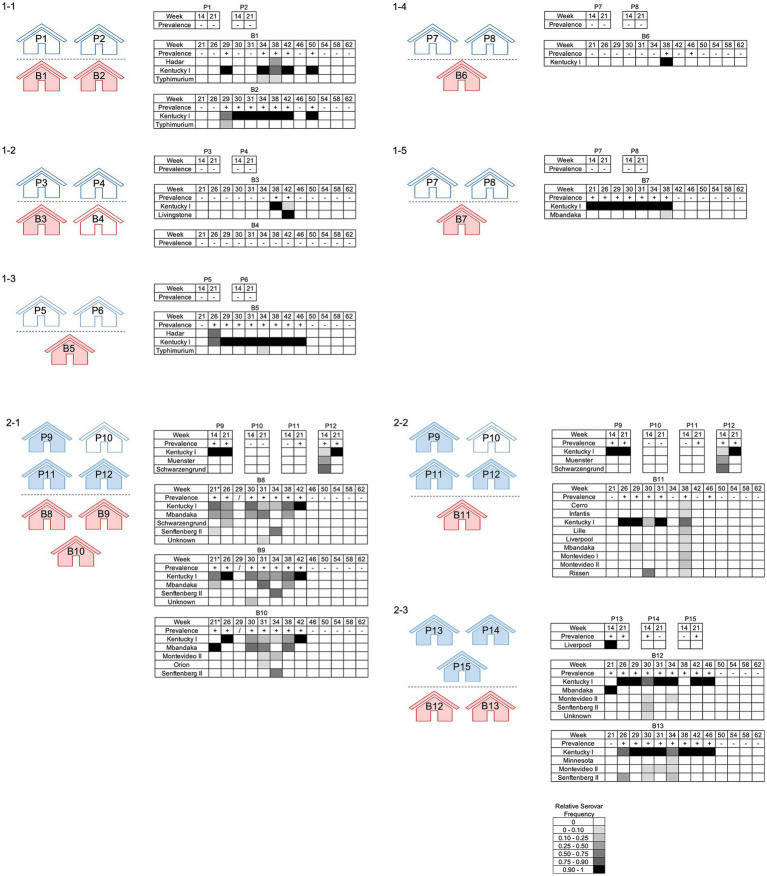
*Salmonella* prevalence and serovar distribution in pullet and breeder flocks across two complexes. Pullet (blue) and breeder (red) houses are shown; *Salmonella-*positive houses are indicated by shading. The breeder flocks that originated from shared pullet flocks are indicated by the numbering. The prevalence indicates whether or not a house was positive (+) or negative (−) for *Salmonella* on the corresponding sampling week. The relative serovar frequencies are reported as determined by deep serotyping via CRISPR-SeroSeq. *The houses on Complex 2 Farm 1 (2–1) had birds placed the morning of week 21 so the houses were not empty during sampling, but they were cleaned out prior.

Deep serotyping of breeder flocks detected five serovars in Complex 1 and 15 serovars in Complex 2 (Figure 6; *p* < 0.00005, Shannon diversity index with Hutcheson t-test). There was a maximum of nine serovars detected from one flock (B11, week 38), with an average of 1.6 serovars per sample. Four serovars were found in pullet flocks from Complex 2. Two out of the four serovars (Kentucky and Schwarzengrund) identified in the pullets were also found in the corresponding breeder flocks. In pullets, 18% (2/11) of boot socks contained more than one serovar., while 38% (51/135) of boot socks from breeders had multiserovar populations. Serovar Kentucky was the most predominantly identified serovar from the breeder flocks, being detected in all (n = 12) breeder flocks that were *Salmonella*-positive. Serovar Mbandaka was also frequently detected (5/6 flocks from Complex 2). Of note is that serovar Mbandaka was only detected in one source pullet flock (P13). Across the two complexes, serovar complexity was highest in samples collected during weeks 30, 31, 34, and 38 (Figure 5B; *p* = 0.02, Fisher’s Exact Test). Collectively, these data demonstrate that *Salmonella* serovar diversity differs between complexes and management strategies, namely integrated pest control, and *Salmonella* surveillance could be optimized around 34–38 weeks.

**Figure 7:**
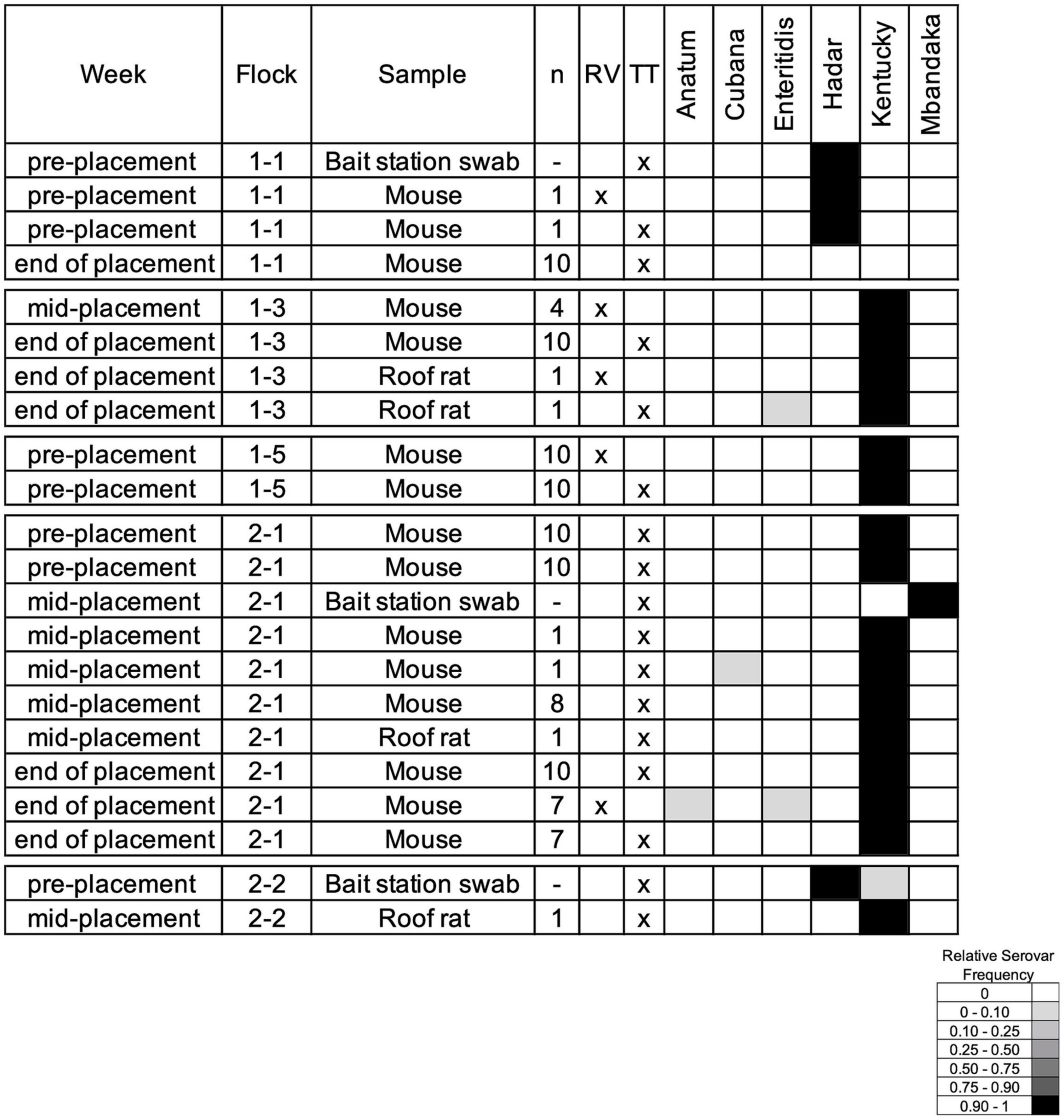
*Salmonella* serovars isolated from rodent composite samples and bait station swabs. The week column corresponds to the flock age when sampling, n includes how many individual rodents comprised the composite sample, and the RV/TT columns indicate the selective media that *Salmonella* was recovered from. The relative serovar frequencies are reported as determined by deep serotyping via CRISPR-SeroSeq. Three of the identified serovars were also found in the corresponding flock samples.

To evaluate whether *Salmonella* transmission occurs between rodents and breeder flocks, we tested rodents collected on the breeder farms, both inside and outside the houses. During the production cycle and immediately following farm depopulation, the gastrointestinal (GI) tract of 355 rodents (49 composite samples with a maximum of 10 GI tracts included for individuals of the same species that were captured from the same house) were cultured for *Salmonella*, along with 33 bait station swabs. House mice provided the majority of GI tracts (300/355; 38/49 composite samples), followed by roof rats (46/355; 8/49), and Norway rats (9/355; 3/49). In total, 35% (17/49) of composite samples and 9% (3/33) of bait station swabs were *Salmonella*-positive, and six serovars were identified (Figure 7). None of the Norway rats were positive for *Salmonella*, while 50% (4/8) of the roof rat and 34% (13/38) of the house mice composite samples were positive. As observed within the breeder flocks, serovar Kentucky was most often present within the rodent samples as well. Serovar Mbandaka was only recovered in the bait station swabs although it was also identified in the boot socks collected from the breeder flocks. Interestingly, serovars Anatum, Cubana, and Enteritidis were isolated exclusively from rodents and not any flock samples, demonstrating that external factors may influence cyclical transmission and rodent populations can introduce *Salmonella* to breeder flocks.

## Discussion

This study, to our knowledge, is the first to characterize changes in multiserovar populations over time in breeder flocks and also to document significant patterns of *Salmonella* serovar co-occurrence in any animal production system. About 20% (104/568) of breeder flocks from this study contain multiple *Salmonella* serovars, which demonstrates the need for routine surveillance to identify all serovars present to properly assess the risk and apply mitigation strategies. Our previous work (Siceloff et al., 2022) with a subset of samples from the GPLN dataset found 32% (43/134) contained multiple serovars; this difference in multiserovar populations may be attributed to the four-fold increase in the number of isolates/samples in the dataset which may have led to a decrease in multiserovar prevalence but overall increase in serovar diversity, as the current study identified 38 serovars while the previous found 26 serovars. Serovar diversity may still be underrepresented in this study as we only analyzed one boot sock collected from each flock. Other work, albeit in broiler flocks, not breeders, has demonstrated the need to collect two boot sock pairs for a more complete understanding of the *Salmonella* population dynamics (Obe et al., 2023). In that study, which began after we started the current study, it was noted that a single boot sock pair from a broiler house was not always sufficient to capture the full serovar diversity in a single house because in 33% of instances, deep serotyping data from a second boot sock pair contained another serovar. Here, in our 15-house longitudinal study, 20% of instances required two boot socks. Nonetheless, deep serotyping identifies more serovars than isolated by culture alone (38 serovars with CRISPR-SeroSeq vs. 32 serovars with colony picking in 568 GPLN samples) and so provides a better idea of the complexity of serovar ecology within our dataset.

From surveillance sampling through GPLN, 35% of breeder flocks were *Salmonella*-positive, while 43% of samples from the longitudinal study were positive. Since GPLN receives the most samples around 16 and 42 weeks, the resulting prevalence may be an underestimation as samples from flocks around the peak and late age classes are not submitted as often. Together, the overall prevalences are comparable to a longitudinal study conducted in Australia, where 36% of breeder flocks were *Salmonella*-positive but higher than the prevalence observed from breeder flocks in Ontario, Canada (25%)(Murray et al., 2023; Willson and Chousalkar, 2023). Our results differ from the Australian study with regards to peak *Salmonella* prevalence as they found their highest number of positive samples at week 7. These results may differ due to geography and different management and production practices between the United States and Australia. Further, serovar profiles as detected by deep serotyping may not be wholly reflective of native *Salmonella* populations as selective enrichment is required prior to sequencing and may promote media bias. Previous work has demonstrated that media bias exists, such that some serovars may be preferentially enriched in one medium when compared to another, and this may be partially overcome by the use of multiple enrichment media (Gorski et al., 2024). In this study, only tetrathionate (TT) broth was used in culturing the breeder boot sock samples since we opted to follow industry standards, as prescribed by NPIP, and so, we acknowledge that the resulting serovar profiles may be skewed.

Our results from GPLN show that *Salmonella* prevalence is highest during peak production (28–35 weeks), while our longitudinal study, which was limited to two complexes, suggest that peak prevalence occurs at 38 weeks. Increased *Salmonella* prevalence may be observed around this timeframe due to the increased stress that birds as experiencing as the most eggs are laid during peak production and this is hypothesized to correlate with greater shedding. Another difference in the two studies presented here is that in the GPLN data, 36% (286/800) of flocks older than 50 weeks were positive for *Salmonella*, while in the longitudinal study, none of the flocks were positive after 50 weeks. Additionally, some serovars were more abundant in the GPLN dataset when compared to the longitudinal study, including the frequent identification of both serovars Cerro and Mbandaka. Therefore, while this study demonstrates that broad surveillance approaches can generate strong trends with respect to *Salmonella* prevalence, integrators should consider that their complexes may differ in terms of determining the peak shedding period and the serovar profiles. This is important since optimizing *Salmonella* surveillance can lead to the development of targeted management approaches, such as vaccination. Nonetheless, the GPLN data, which represents 22 different companies, suggests that monitoring *Salmonella* during peak production would be more helpful than the current time at 42 weeks.

The prevalence of and the interactions between serovars Cerro and Mbandaka from the GPLN dataset was an unexpected result, as they are commonly found in cattle but not in broilers. Between 2016–2023, FSIS found serovars Cerro and Mbandaka in 0.045% (4/8853) and 0.21% (19/8853) *Salmonella*-positive broiler samples, respectively, and none originated from facilities in Georgia. A recent study in four broiler complexes found low prevalence of these serovars; serovar Cerro was found in one of 68 positive houses, and serovar Mbandaka in four of the houses, including the same house where Cerro was detected (Obe et al., 2023). Therefore, beyond breeders, the incidence of these two serovars in poultry production and processing is significantly reduced. There are three potential explanations for this. First, it is possible that these serovars are entering in contaminated feed as meat and bone meal are a common ingredient for chickens, and this would explain the presence of cattle-associated serovars since multiple animals may be included in the ground product. To address this, producers could monitor *Salmonella* in feed. Breeders are typically fed a mash diet, while broilers are fed a pelleted diet which has an additional pathogen reduction step as extrusion is required during the pelleting process and thus may explain the lower incidence in broilers. However, this also opens the possibility that these serovars may not be present in the birds themselves (or may not be actively shed), and that the industry standard of environmental boot sock sampling leads to the detection of *Salmonella* in feed that has fallen on the slats/floor. The serovar profile observed in house B11 at week 38, which includes serovars Mbandaka and Cerro, as well as serovar Rissen (most often found in swine), may exemplify the occurrence of atypical chicken *Salmonella* serovars in breeder feed. At the following sampling (42 weeks), the house was *Salmonella-*negative. The second potential explanation is that some poultry growers also have cow-calf operations so this practice may serve as a potential entry source for these serovars. Given that the co-occurrence of serovars Cerro and Mbandaka was observed in 19 different flocks, rather than limited to a few farms, we think this is unlikely to be a significant introduction event. The third explanation is that the application of *Salmonella* vaccines in breeders is suppressing specific serovars (i.e., serovars Typhimurium, Enteritidis, and Infantis) and that this provides the opportunity for less competitive serovars such as Cerro and Mbandaka to colonize breeders. Where vaccine pressure is subsequently reduced in broilers, these serovars could then be replaced by those that are better adapted to poultry. Vaccine pressure could also explain the low incidence in our study of serovars that are often found at processing, including Typhimurium (4.4%; 25/568), Enteritidis (1.4%; 8/568), and Infantis (3.3%; 19/568). For example, during the same time frame, serovar Infantis was found in 18% (11/61) and 23% (53/226) of regulatory carcass and parts samples, respectively (Figure 2). Alternatively, or in combination with vaccine pressure, the increased presence of serovar Infantis in broiler carcasses and parts may be due to increased survival during the antimicrobial interventions at processing, though a recent study did not find evidence of this occurrence (Richards et al., 2024). Serovar Schwarzengrund is interesting as it was not found at appreciable frequency in breeders, but accounts for 11 and 17% of carcasses and parts, respectively, from Georgia. We hypothesize that, being an O:4 (Group B) serovar., it may be inhibited by the serovar Typhimurium live vaccine.

There has been a substantial amount of work conducted to explore the physiological traits of select serovars of animal or human clinical importance, namely serovars Enteritidis and Typhimurium. However, studying the growth dynamics within multiserovar populations are a more recent consideration. In one elegant study, the fitness of two serovars (Kentucky and Typhimurium) did not differ when grown individually in chicken cecal contents. Rather, limited growth of serovar Typhimurium only manifested when co-cultured alongside serovar Kentucky (Cheng et al., 2015). Further, a cell invasion-deficient serovar Kentucky strain did not have reduced colonization in chickens compared to a cell invasion-proficient strain of serovar Typhimurium, which supports the finding that differential growth rates in host can be driven by stress response pathways rather than virulence factors (Cheng et al., 2015). An additional study found that serovar dominance in mixed populations may simply be dependent on which serovar colonized the host first (Yang et al., 2018). Competitive exclusion has been utilized to inhibit *Salmonella* colonization in poultry production, but additional work is required to characterize this phenomenon in multiserovar populations and identify the driving forces in serovar dominance (Soerjadi et al., 1981; Nisbet et al., 1998; Nava et al., 2005; Bailey et al., 2007; Methner et al., 2011; Micciche et al., 2018; Bucher et al., 2020; Pineda et al., 2021; Maurer et al., 2024). Similarly to other control strategies, such as vaccination, competitive exclusion may have unexpected consequences as the removal of one serovar from a system leaves an open niche for another, potentially higher risk, serovar (Rabsch et al., 2000; Foley et al., 2011).

There are opportunities for pathogen colonization at each stage of poultry production, as the individual components of feed could be contaminated and distributed among farms, eggs with excess fecal content could spread pathogens from farm to hatchery, or any lapse in on-farm biosecurity could serve as an introduction event (Dale et al., 2015; English et al., 2015; Rajan et al., 2017; Vinueza-Burgos et al., 2019; Machado Junior et al., 2020; Wang et al., 2023). We observed similar serovar profiles between multiple houses on one farm, as 70% (90/129) of farms contained similar populations (Bray-Curtis: 0–0.3), emphasizing the need to ensure that on-farm biosecurity is promoted to prevent *Salmonella* introduction and transmission. Rodents may act to introduce *Salmonella* to flocks since they are known to be vectors, and this observation was supported in our study as we found several matching serovars between rodent and breeder boot sock samples. However, further characterization to the strain level is required to confirm transmission in this study. One previous study isolated the same strain of serovars Enteritidis and Typhimurium (Liljebjelke et al., 2005) between rodents and flocks, which underscores the importance of pest control on farm toward reducing *Salmonella* in the flocks. There have been limited studies conducted on the role of rodents in on-farm *Salmonella* transmission, and most have focused on layer flocks, so future work is required to understand the impact of rodents upon *Salmonella* diversity (Henzler and Opitz, 1992; Davies and Wray, 1995; Guard-Petter et al., 1997; Garber et al., 2003; Meerburg and Kijlstra, 2007; Lapuz et al., 2012; Guard et al., 2018; Camba et al., 2020).

We noted higher *Salmonella* prevalence and greater serovar complexity in breeder flocks that were positive as pullets. This may indicate that early monitoring and response in pullets is a good strategy for reducing *Salmonella* in breeders. Since we collected noninvasive environmental samples, there is a possibility that these serovars were present in the pullet flocks but remained undetected due to low quantity or lack of shedding at the time of sampling. However, since we began sampling after the pullets had been in the houses for 14 weeks, we would expect to find evidence of *Salmonella* colonization in the litter. As the longitudinal study was only across two complexes, further studies would be needed to confirm the impact of *Salmonella* incidence from pullets to breeders.

While this study only included breeder flocks from the southeast, the framework presented here provides support to develop robust *Salmonella* surveillance at any stage of live production. Further, the findings can be broadly applicable to the domestic poultry industry as Georgia contributes 14% of broilers to the national poultry production, which is the greatest amount by any one state and the eighth highest when comparing annual chicken production volumes by countries (United States Department of Agriculture - National Agriculture Statistics Service, 2023; United States Department of Agriculture - Foreign Agricultural Service, 2024). The observed population dynamics demonstrate that select serovars can impact the presence of others, underscoring the importance of future work to explore interserovar relationships and physiological mechanisms behind competitive exclusion of serovars. To that end, this study also demonstrates the need for high-resolution surveillance approaches, as characterizing serovar interactions and developing targeted solutions requires the reliable and robust detection and relative quantification of all present serovars. The pre-harvest reduction of *Salmonella* in of all types of food animal production systems supports further reductions at processing, so it is critical to understand the driving factors behind population dynamics in food animal production and enact effective control strategies. The framework presented here can be applied to other food animal production systems where *Salmonella* is a problem. Finally, amplicon-based approaches can be extended to other infectious organisms that occur in mixed populations to investigate relationships among bacterial subtypes or among viral variants.

## Data Availability

The sequence data has been uploaded to NCBI SRA as part of BioProject PRJNA1204137.

## Footnotes

^1^http://www.bioconductor.org/packages/release/bioc/html/DESeq2.html
