# Supplementary figures and images for: Longitudinal study highlights patterns of Salmonella serovar co-occurrence and exclusion in commercial poultry production
Source: Front Microbiol. 2025 Jul 16;16:1570593. doi: 10.3389/fmicb.2025.1570593 (PMC12307445; doi:10.3389/fmicb.2025.1570593)

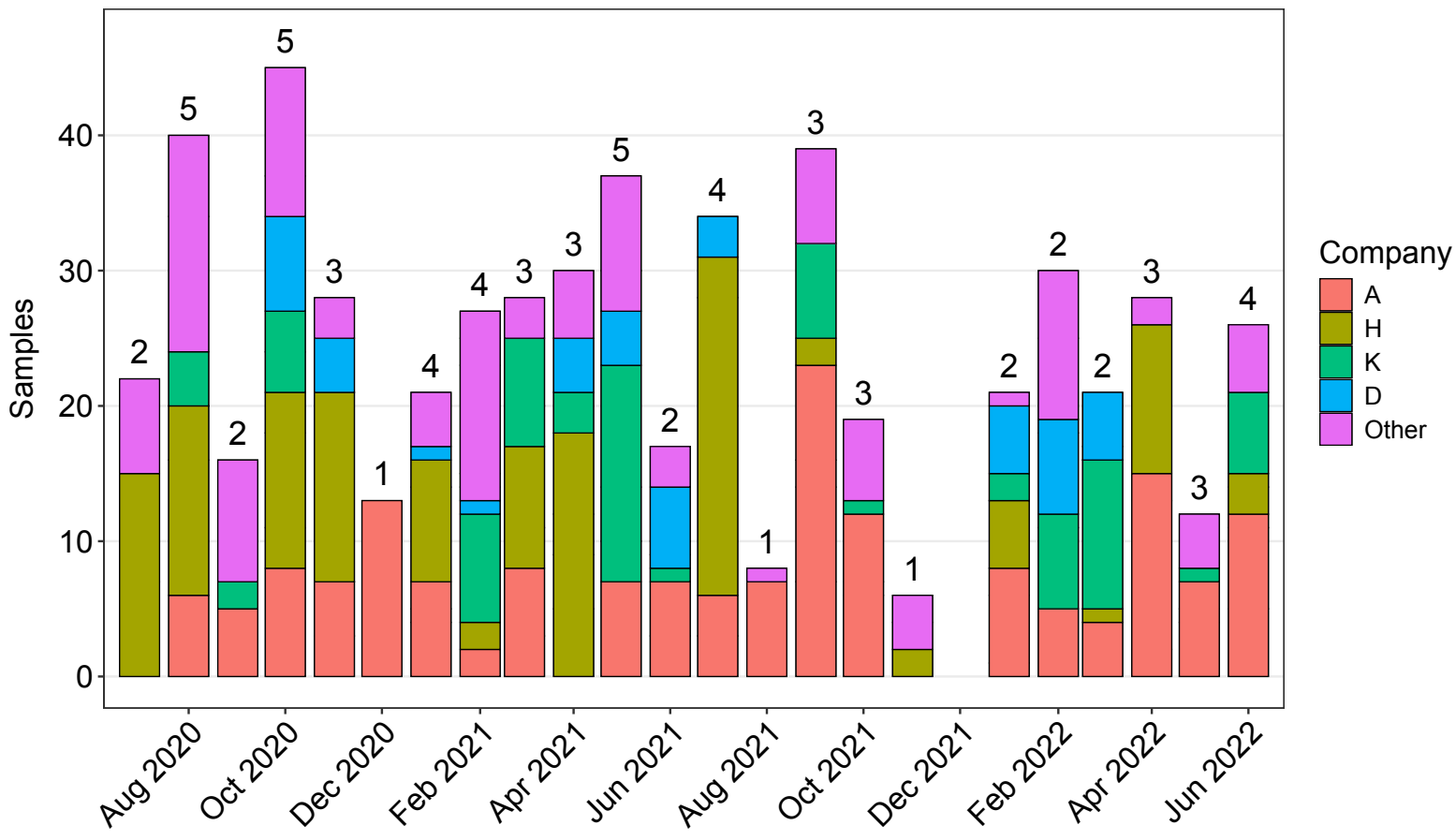

Supplement: Supplementary file 1 [file Image_1.PDF]

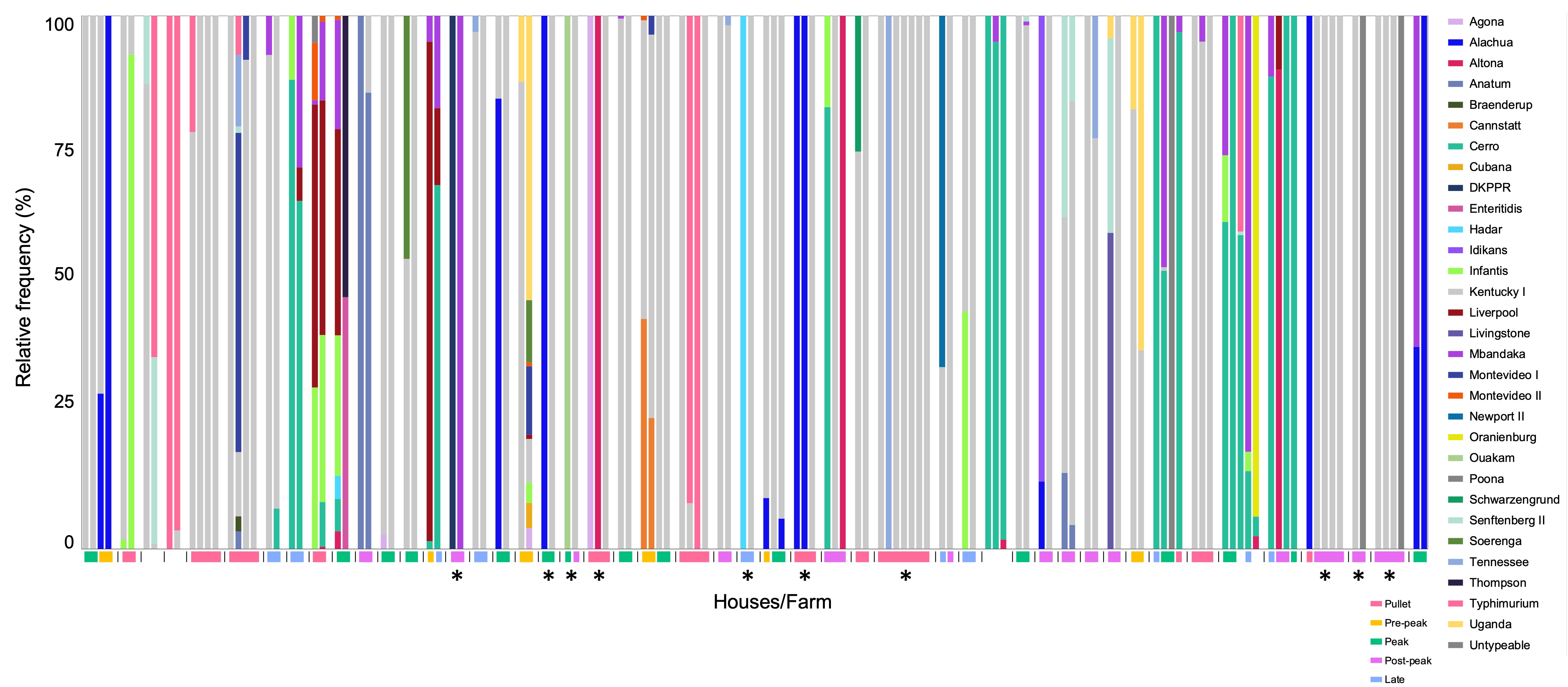

Supplement: Supplementary file 2 [file Image_2.JPEG]

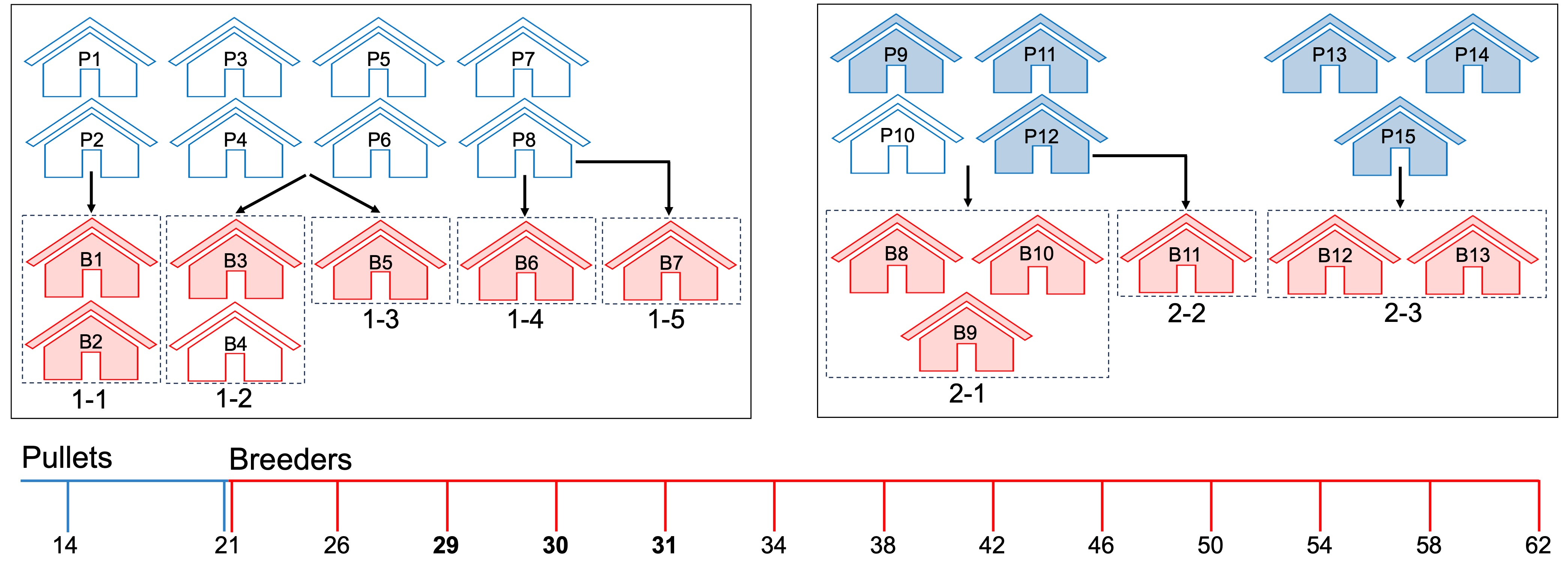

Supplement: Supplementary file 3 [file Image_3.JPEG]
